# A comparative study of intrathecal and epidural buprenorphine using combined spinal-epidural technique for caesarean section

**DOI:** 10.4103/0019-5049.65359

**Published:** 2010

**Authors:** Shaloo Ipe, Sara Korula, Sreelatha Varma, Grace Maria George, Saramma P Abraham, Leena Rachel Koshy

**Affiliations:** Department of Anaesthesiology, MOSC Medical College, Kolenchery, Kerala, India; 1 Department of Anaesthesiology, Malabar Institute of Medical Sciences, Kozhikode, Kerala, India

**Keywords:** Buprenorphine, caesarean section, epidural, intrathecal

## Abstract

Neuraxial opioids provide excellent analgesia intraoperatively and postoperatively while allowing early ambulation of the patient by sparing sympathetic and motor nerves. A prospective, randomised double blind study was conducted involving 90 patients of ASA 1 physical status coming for elective cesarean section to evaluate the analgesic effect of neuraxial buprenorphine. They were allocated into three groups. Spinal local anaesthetic was used as the main stay of anaesthesia for surgery and spinal and epidural analgesia with opioids continued as the main stay for postoperative analgesia. All the groups were given 0.5% Bupivacaine intrathecally for the surgery. Besides this, group I was given 150 mcg Buprenorphine intrathecally and group II and III were given 150 mcg and 300 mcg Buprenorphine respectively, epidurally. In the present study, we observed that 150 mcg of Buprenorphine given intrathecally provided much longer duration of analgesia compared to 150 mcg of Buprenorphine given epidurally. Increasing the epidural dose of Buprenorphine from 150 mcg to 300 mcg proved to produce prolonged analgesia comparable to intrathecal Buprenorphine without compromising patient safety and neonatal outcome. The minor side effects were more with intrathecal Buprenorphine than epidural Buprenorphine. We concluded that 300 mcg of Buprenorphine epidurally is equianalgesic to 150 mcg Buprenorphine intrathecally.

## INTRODUCTION

Opioids are widely used for providing postoperative analgesia and advantages of neuraxial narcotics over systemic narcotics are well established.[1] Opioids, when compared to local anaesthetics, offers the advantage of providing good analgesia while allowing early ambulation of the patient by sparing sympathetic and motor nerves.[2] Buprenorphine is a long acting, highly lipophilic opioid, which has proved to be a promising analgesic, by epidural and intrathecal route.[3,4] It is about 25 times more potent than morphine and has a low level of physical dependence.[5]

There are numerous studies comparing opioid agents in varying concentrations given through either epidural route or intrathecal route separately. But there are few studies comparing the effects of the same opioid given intrathecally and epidurally and few studies on analgesic effects of neuraxial buprenorphine.

The present study is undertaken to compare the effects of intrathecal and epidural buprenorphine and to find a safe equianalgesic dose of intrathecal and epidural Buprenorphine. Quality and duration of analgesia was assessed for 24 hours postoperatively. Occurrence of adverse effects like hemodynamic effects, respiratory depression, Post Dural Puncture Headache, Nausea and vomiting, drowsiness and pruritus when given through each of the routes were also assessed. Neonatal outcome was evaluated using Apgar score[6] and neonatal umbilical cord pH.[7] Patient’s and surgeon’s acceptability of the technique was noted.

## METHODS

A randomised controlled double blind prospective study was done to compare the effects of intrathecal and epidural Buprenorphine. The study was conducted after approval by the hospital Ethics Committee and an informed written consent was obtained from all the patients. A total number of 90 ASA I patients belonging to age group 20-40 years posted for elective lower segment cesarean section were divided into three groups of 30 each. Group allocation was achieved by a computer generated randomisation list. Patients with any variations from normal were excluded.

The patients were kept fasting for 6 hours prior to surgery and premedicated with oral Ranitidine 150 mg at night and oral Metoclopramide 10 mg and Ranitidine 150 mg two hours prior to the surgery. NIBP, ECG, SPO2, RR were monitored up to 24 hrs postoperatively. An 18g IV canula was secured and all patients were preloaded with 750 ml of Ringer Lactate before the neuraxial block. Spinal local anaesthetic was used as the main stay of anaesthesia for surgery and spinal and epidural analgesia with buprenorphine continued as the main stay for postoperative analgesia.

A single space Combined Spinal Epidural technique was chosen and the same volume of drug was injected intrathecally and epidurally in all study groups. To make the intrathecal volume of drug equal in all groups, group II and III were given 0.5% Bupivacaine 2.5 ml intrathecally while group I was given 0.5% Bupivacaine 2 ml and 0.5 ml of buprenorphine (a total of 2.5 ml)

Grouping of cases was in the following manner:-

**Table:** 

Intrathecal	Epidural
Gp I : 2ml of Bupivacaine 0.5% with 0.5 ml Bupinorphine (150mcg)	3 ml of 2% xylocaine with adrenaline(15mcg) and 7ml Normal saline
Gp II: 2.5 ml Bupivacaine 0.5%	3 ml of 2% xylocaine with adrenaline( 15mcg) and 6.5ml of Normal Saline + 0.5ml Bupinorphine (150mcg)
Gp III : 2.5 ml Bupivacaine 0.5%	3 ml of 2% xylocaine with adrenaline( 15mcg) and 6 ml of Normal Saline + 1ml Bupinorphine (300mcg)

The procedure was carried out in lateral decubitus position using combined spinal epidural needle. Epidural space was identified in L2-L3 region using loss of resistance technique. The anaesthetist conducting the study was blinded to the study drug which was prepared by another anaesthetist as per instructions. Epidural test dose of 3 ml of xylocaine with adrenaline was given and observed for any motor block or significant rise in heart rate. After injecting 7 ml of the epidural test drug, spinal needle was advanced into the subrachanoid space and 2.5 ml of intrathecal test drug was given. A left uterine displacement of 15° was maintained during surgery. Supplemental oxygen was given through a poly mask.

Three groups were compared.

**Figure 1:**
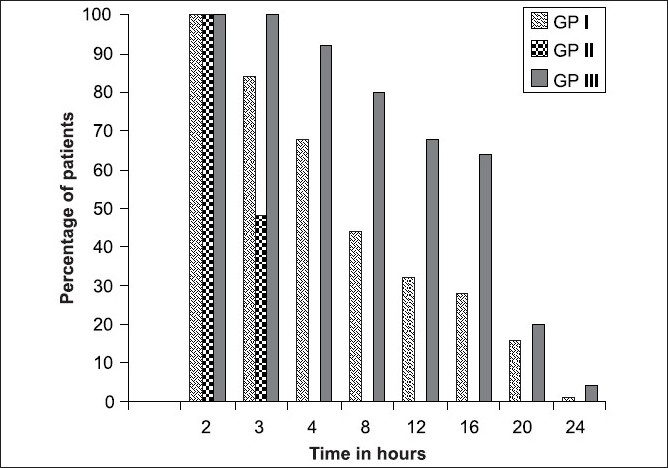
Duration of analgesia

Sensory block was tested by pinprick till level reached T4. The total duration of analgesia was calculated from onset of sensory block to end of analgesia i.e.; pain score of 5 or more on the Verbal Numerical Rating Scale [Figure 1].[8]

Verbal numerical rating scale

**Table:** 

Pain score	Degree of pain	Degree of analgesia
0	No pain	Profound Analgesia
2-4	Mild pain	Moderate Analgesia
5-7	Moderate pain	Mild Analgesia
8-10	Worst pain	No Analgesia

Criteria for hypotension was taken as a systolic BP less than 100 mm Hg and bradycardia as heart rate less than 60. Hypotension was treated with IV ephedrine and rapid infusion of fluid. Criteria for respiratory depression were a fall in oxygen saturation to <90%, a respiratory rate less than 12/min. At the end of the surgery, the overall quality of anaesthesia was judged by the surgeon and patient on a Numerical Rating Scale (NRS) from 1 (unsatisfactory) to 10 (excellent). Neonatal outcome was assessed by APGAR score at I min and 5 min and by umbilical arterial blood gas analysis. APGAR score of <7 and an umbilical arterial blood pH <7.2 was considered abnormal.

Postoperatively patients were monitored in post anaesthesia care unit and complications like hypotension, respiratory depression, drowsiness, post dural puncture headache, nausea, vomiting and pruritus were assessed for 24 hours. Occurrence of urinary retention could not be assessed as the patients for LSCS are routinely catheterised for 24 hours postoperatively in our institution.

### Statistical analysis

Parametric data were analysed using Z test. *P* < 0.05 was considered as significant.

## RESULTS

In all the three groups, there was no significant change in heart rate and blood pressure from the base line value, neither in intraoperative nor in the postoperative period (*P*>0.05). The minimum blood pressure recorded was systolic BP of 84 mm Hg and fall in BP was transient and responded to one dose of 6mg ephedrine IV. There was no significant variation in respiratory rate and saturation in all three groups and were within acceptable limits [Table 1].

**Table 1:** Demographic and other data (mean ± SD)

	Group I	Group II	Group III
Age (Years)	24.5 ± 3.38	23.7 ± 2.07	24.1 ± 3.28
Weight (kg)	67.77 ± 5.29	69.01 ± 4.71	66.27 ± 4.6
Height (cm)	155.7 ± 5.3	155.3 ± 5.6	155.6 ± 4.01

Incidence of nausea and vomiting was 20% in group I and 16% in group II and III. Incidence of pruritus was 20% in group I, 4% in group II and 16% in group III. No patient had post dural puncture headache, backache or drowsiness in any group [Table 2].

**Table 2:** Complications and side effects

Groups	Max. fall in systolic BP	RR	SpO^2^	Change in heart rate	Nausea and vomiting (%)	Pruritus (%)
I	19.76 ± 7.38	17 ± 1.5	98.64 ± 0.40	12.73 ± 2.91	20	20
II	19.88 ± 5.40	17.2 ± 1.3	98.64 ± 0.49	13.07 ± 3.65	16	4
III	19.36 ± 6.5	16.8 ± 1.00	98.43 ± 0.61	12.56 ± 3.01	16	16
*P* value						
I vs. II	*P* = 0.94	*P* = 0.58	*P* = 1	*P* = 0.68	*P* = 0.68	*P* = 0.048
	NS	NS	NS	NS	NS	S
I vs. III	*P* = 0.82	*P* = 0.54	*P* = 0.114	*P* = 0.82	*P* = 0.68	*P* = .0.68
	NS	NS	NS	NS	NS	NS
II vs. III	*P* = 0.73	*P* = 0.18	*P* = 0.1416	*P* = 0.55	*P* = 1	*P* = 0.109
	NS	NS	NS	NS	NS	NS

The 1 minute and 5 minute Apgar score and the umbilical cord pH of babies in all the three groups were acceptable and there was no significant differences between the groups (*P*>.05) [Table 3].

**Table 3:** Neonatal assessment

Groups	Umbilical pH	APGAR score 1 min	APGAR score 5 min
I	7.29 ± 0.04	8.32 ± 0.75	9.36 ± 0.49
II	7.27 ± 0.08	8.4 ± 0.76	9.44 ± 0.51
III	7.28 ± 0.02	8.44 ± 0.51	9.44 ± 0.52
*P* value			
I vs. II	*P* = 0.22	*P* = 0.68	*P* = 0.53
	NS	NS	NS
I vs. III	*P* = 1.22	*P* = 0.47	*P* = 0.54
	NS	NS	NS
II vs. III	*P* = 0.66	*P* = 0.81	*P* = 1
	NS	NS	NS

In all groups the surgeon enjoyed adequate muscle relaxation for performing the surgery and patient was comfortable throughout the procedure. There was no statistically significant difference between the groups in the NRS scores [Table 4].

**Table 4:** Surgeon’s and patient’s acceptability of anaesthesia

Groups	NRS* surgeon	NRS patient
I	9.2 ± 1.4	8.8 ± 2.6
II	9.0 ± 1.2	8.4 ± 1.2
III	9.1 ± 2.4	9.0 ± 2.4
*P* value		
I vs. II	*P* = 0.552	*P* = 0.77
	NS	NS
I vs. III	*P* = 0.84	*P* = 0.31
	NS	NS
II vs. III	*P* = 0.84	*P* = 0.225
	NS	NS

* NRS - Numerical rating scale

### Time in hours

In group I (intrathecal 150 mcg Buprenorphine), 100% of patients had analgesia till 2.5 hours and 50% had analgesia till 6 hours. At 20 hours and 24 hours 16% and 1% of patients, respectively, had analgesia.

In group II (epidural 150 mcg Buprenorphine) 100% of patients had analgesia till 2 hours and 50% till 3 hours. Thereafter the analgesia was poor.

In group III (epidural 300 mcg Buprenorphine) till 3.5 hours 100% of patients had analgesia and it took 16 hours for the percentage to drop to 50%. At 20 hours and 24 hours, 20% and 4% of patients, respectively, had analgesia.

## DISCUSSION

Any method of postoperative analgesia must meet three basic criteria; it must be simple, safe, clinically appropriate and evidence based.[9] The majority of postoperative patients managed with parenteral or intramuscular opioid drugs are left with unrelieved pain.[10,11] The discovery of opioid receptors in the brain and spinal cord started a new era in the field of postoperative analgesia.[12,13]

Buprenorphine is a mixed agonist – antagonist type of opioid with a long duration of action. The high lipid solubility; high affinity for opioid receptors and prolonged duration of action makes Buprenorphine a suitable choice for intrathecal and peripheral nerve site administration.[3,4]

Addition of Buprenorphine 150 mcg intrathecally or epidurally and 300 mcg epidurally provided good postoperative analgesia without prolonged motor block.[14,15] In all three groups, 100% patients had analgesia till two hours. 50% of patients had analgesia up to 6 hours in group I, 3 hours in group II and 17 hours in group III. Duration of analgesia was poor with 150 mcg Buprenorphine epidurally. The mean duration of analgesia was highest in Group III with 300 mcg Buprenorphine epidurally, 4% of patients had analgesia till 24 hours. Subarachnoid and epidural 150 mcg Buprenorphine are not equi-analgesic. When epidural dose of Buprenorphine was increased from 150 mcg to 300 mcg, analgesia improved considerably. This confirms the observation that lipophilic opioids need higher doses epidurally to be effective.[16,17]

One of the main concerns with Buprenorphine is respiratory depression.[18–20] In our study, none of the patients in the study groups had respiratory depression. Arterial oxygen saturation in all the cases remained above 96% and mean respiratory rate of all patients were above 17. None of the patients required any respiratory support. The mean fall in BP was comparable in all groups and hypotension if present was transient. The blood pressure and heart rate were acceptable in all groups. This was similar in earlier studies.[21]

The incidence of Nausea and Vomiting was 20% in GP I which was slightly higher than the other groups. Pruritus is one of the commonest side effects of neuraxial opiods. It is more likely to occur in obstetric patients due to the interaction of estrogen with opioid receptors. Previous studies show the incidence of pruritus after epidural administration of 50 mcg fentanyl was 47% and with 300 mcg Buprenorphine, 10%.[22] In our observation, pruritus of a mild nature occurred in all three groups but was slightly higher with subarachnoid Buprenorphine than epidural groups.

Incidence of PDPH, backache and drowsiness were not reported. This was comparable to the results obtained by Fuller JG *et al*.,[23] and Escarment J *et al*.,[21] The high rate of nausea and pruritus in these patients warrants the use of ondansetron for premedication and for at least 24 hours postoperatively till the effect of opioids wear off.[24]

Neonatal outcome was good in all the groups as assessed by 1min and 5 min Apgar and umbilical arterial blood pH.[25] In all patients the anaesthesiologist and surgeon found the anaesthesia to be adequate for the operative procedure in terms of pain relief and relaxation. All patients were comfortable and willing to accept the same anaesthetic technique for a similar procedure in future.

One of the drawbacks of the study was that the latency of onset of action of Buprenorphine could not be studied because it was masked by the action of subarachnoid local anaesthetic. Also the concentration and quantity of intrathecal local anaesthetic in Group I was dilute (2.0 ml of Bupivacaine and 0.5 ml of Buprenorphine). Urinary retention could not be assessed because in our institution all patients for caesarean section are catheterised for 24 hours postoperatively.

## CONCLUSION

1. Buprenorphine 150 mcg intrathecally, 150 mcg epidurally and 300 mcg epidurally provided good quality postoperative analgesia.
2. 150 mcg Buprenorphine epidurally was not equianalgesic to 150 mcg Buprenorphine given intrathecally. The duration of analgesia was much shorter with epidural 150 mcg Buprenorphine.
3. Increasing the epidural dose of Buprenorphine from 150 to 300 mcg, prolonged postoperative analgesia up to 24hours without compromising patient safety and neonatal outcome.
4. The minor side effects of Buprenorphine are more when administered intrathecally, compared to Buprenorphine administered epidurally.
